# Prognostic significance of systemic immune inflammation index for ovarian cancer: An updated systematic review and meta-analysis

**DOI:** 10.1186/s13048-025-01626-1

**Published:** 2025-02-27

**Authors:** Boliang Chu, Yingying Chen, Jiewei Pan

**Affiliations:** Department of Gynecology, Huzhou Maternity & Child Health Care Hospital, No. 2, East Street, Wuxing District, Huzhou, Zhejiang Province 313000 China

**Keywords:** Inflammation, Marker, Risk factor, Malignancy, Ovary, Survival

## Abstract

**Objective:**

Several inflammatory indices have been used to assess the prognosis of ovarian cancer, with variable results. This review assessed whether the systemic immune inflammation index (SII) can predict outcomes in patients with ovarian cancer.

**Methods:**

Embase, PubMed, CENTRAL, Web of Science, and Scopus databases were searched by the two reviewers from inception to 15th October 2024 for studies assessing the relationship between SII and overall survival (OS) or disease-free survival (DFS).

**Results:**

Ten studies with eleven cohorts were included. Pooled analysis showed that higher SII was a significant predictor of poor OS (HR: 2.35 95% CI: 1.56, 3.55 I^2^ = 88%) and worse DFS (HR: 2.51 95% CI: 1.71, 3.67 I^2^ = 80%) after ovarian cancer. Sensitivity analysis failed to change the significance of the results. No publication bias was noted. Most results remained significant on subgroup analyses based on location, sample size, FIGO stage, treatment, adjusted outcomes, cut-off of SII, method of determining cut-off, and quality score.

**Conclusions:**

SII can be a potential predictor of OS and DFS after ovarian cancer. Further studies are required to improve the evidence.

**Supplementary Information:**

The online version contains supplementary material available at 10.1186/s13048-025-01626-1.

## Introduction

Ovarian cancer is the eighth most common female cancer, accounting for about 313,959 new diagnoses and 207,252 cancer-related deaths worldwide in 2020 alone [1]. As cancer progresses asymptomatically in most cases, the detection is usually done in advanced stages, leading to poor prognosis [2]. Despite recent advances in diagnosis and treatment protocols in the past decade, the five-year survival rate of Stage III or IV ovarian cancer is only 45% [3, 4], and the median overall survival (OS) and disease-free survival (DFS) after surgical intervention are about 33.9 and 10.7 months, respectively [5]. Given the poor prognosis, a risk stratification strategy based on patient and cancer characteristics is being suggested to predict survival and recognize patients who need more aggressive treatment. Accurate and easily available markers can help formulate individualized surveillance plans for better screening of recurrence [6, 7].

Several systemic inflammatory markers have recently been identified as prognostic markers for oncology patients [8, 9]. The interest in such markers has been generated mainly due to a strong relationship between systemic inflammation and cancer development and progression [10, 11]. The research shows that chronic inflammation aids in cancer growth, vascular proliferation, and metastasis, leading to worse outcomes [12]. While markers like neutrophil–lymphocyte ratio, platelet-lymphocyte ratio, lymphocyte-monocyte ratio, Glasgow Prognostic Score (GPS), modified GPS, systemic immune-inflammation index (SII), and systemic immune response index (SIRI) have all been shown to have a prognostic value, it is still unclear if there is a single marker that is superior to the rest [8, 9].

The SII is a readily available prognostic marker calculated by multiplying the absolute platelet and neutrophil counts and dividing them by the absolute lymphocyte count [13]. This marker has been used to predict survival in several cancers [14–16]. However, evidence for its use for ovarian cancer is limited and conflicting. One reason for the scarce research on ovarian cancer could be the lower incidence of this malignancy, especially when compared to other gynecological cancers [1]. Furthermore, while some studies [17, 18] demonstrate a statistically significant association between high SII and worse outcomes after ovarian cancer, other reports [19, 20] show no such association. While the previous meta-analysis by Mao et al. [21] assessed the prognostic ability of SII for ovarian cancer, it only included six studies. Therefore, a comprehensive meta-analysis is needed to provide robust results. This study aims to present updated evidence on the ability of SII to predict OS and DFS in ovarian cancer.

## Material and methods

This study was reported in line with the Preferred Reporting Items for Systematic Reviews and Meta-Analyses (PRISMA) statement reporting guidelines [22]. The review protocol can be found on PROSPERO's website (CRD42024597854). The protocol was initially registered to examine both the systemic immune-inflammation index (SII) and the systemic immune-inflammation response index (SIRI) as prognostic indicators for ovarian cancer. However, the review was amended only to include SII due to a lack of studies on SIRI.

### Information sources

Embase, PubMed, CENTRAL, Web of Science, and Scopus databases were screened electronically. Two reviewers carried out the search from the database inception up to 15th October 2024 for articles without any restrictions on language and date of publication. Free and MeSH keywords used were ‘ovary’, ‘ovarian’, ‘cancer’, ‘malignancy’, ‘carcinoma’, ‘systemic immune inflammation index’, and ‘SII’. The search strategy of each database can be found in Supplementary File 1. Google Scholar was examined for gray literature, and the reference lists of original included articles, and past reviews were manually searched.

Screening of the search results was carried out independently by the reviewers using a three-tier approach. The first step involved combining and de-duplicating the identified studies using EndNote software (version X9.3.3, Thomson Reuters, Philadelphia, USA). Next, the remaining articles were screened for relevance by reading the titles and abstracts. Relevant studies selected by either reviewer were downloaded for the next phase, and the final selection of studies was done by reading the full texts. Any disagreement was resolved through consensus.

### Eligibility criteria

The inclusion criteria were as follows:

1. Cohort studies, case–control studies, or secondary analysis of randomized controlled trials.
2. Studies reporting the association between SII and outcomes of ovarian cancer.
3. Studies reporting OS or DFS.
4. Studies reporting the effect size of the association.

The exclusion criteria were:

1. Studies not reporting separate data for ovarian cancer.
2. Review articles, unpublished data, non-peer-reviewed studies, case reports, and commentaries.

### Risk of bias

The quality of studies was assessed using the Newcastle Ottawa Scale (NOS) [23]. Two reviewers participated in the assessment, and disagreements were resolved by consensus. Each study was judged on the following domains: participant selection, group comparability, and outcomes. A higher score indicated a better quality of the study.

### Data management

Both reviewers extracted data from the studies using a pre-designed table. Data extracted from all eligible papers consisted of the following items: first author name, year of publication, location, study design, sample size, age, FIGO stage, lymph node metastasis, peritoneal involvement, treatment, cut-off, method of cut-off estimation, follow-up, adjustment of outcomes, and effect size.

The outcome data of interest were OS and DFS. Adjusted data were preferred over raw data. In cases where adjusted data were not reported, univariate data were used for the meta-analysis.

### Statistical analysis

The software used was “Review Manager” (RevMan, version 5.3). Data from individual studies was combined to generate a pooled hazard ratio (HR) with 95% confidence intervals (CI) for both OS and DFS. Effect size data were entered utilizing the generic inverse variance function of RevMan. HR > 1 indicated worse OS/DFS. Heterogeneity among studies was assessed through Cochran’s Q statistic and the *I* ^*2*^ index. *I* ^*2*^ of over 50% and/or *P* < 0.05 indicated a large degree of heterogeneity. Nevertheless, due to the expected variations in study populations, ethnicity, cancer stage, treatment, and follow-up intervals between studies, the inverse variance random-effect model was chosen irrespective of the quantified inter-study heterogeneity. A sensitivity analysis was done by the Review Manager software itself. One study at a time was removed from the meta-analysis to assess the stability of the results. Funnel plots were also generated by Review Manager software to check for publication bias. In addition, Egger’s test was performed for publication bias.

## Results

### Search results

**Fig. 1:**
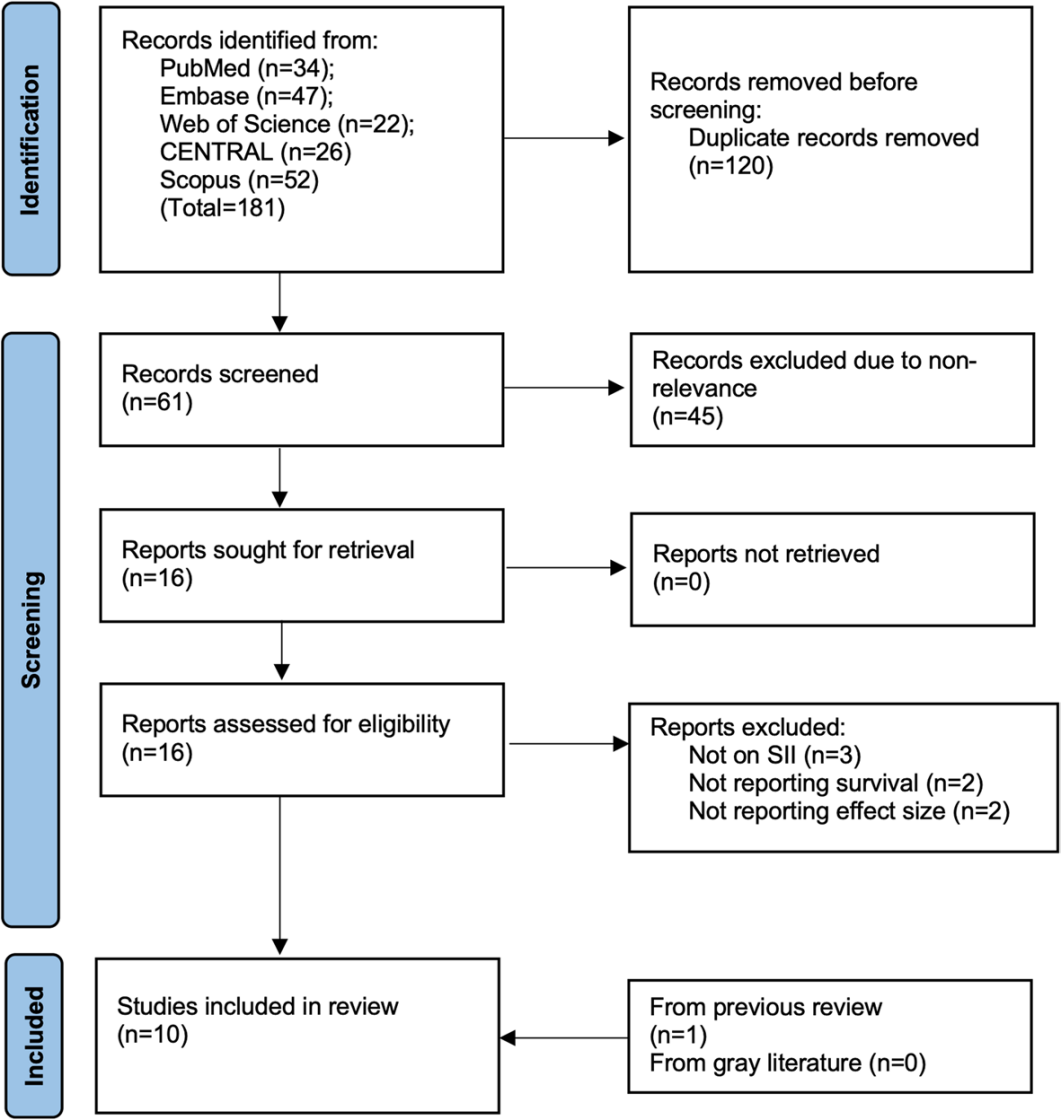
Study flow chart

The number of search results stratified by the stage of the selection process can be found in Fig. 1. The initial screening identified a total of 61 unique studies. Of them, 16 were found to be relevant for further analysis, and nine were included in the final review after full-text analysis. One additional study was found after screening the reference list of previous reviews and included studies. No additional studies were found in the gray literature. Finally, 10 studies [17–20, 24–29] with 11 cohorts were analyzed. A list of excluded studies with reasons for exclusion is presented in Supplementary File 2.

### Details of included studies

Data extracted from the studies is presented in Table 1. Studies were published between the years 2019 and 2024, were retrospective cohort in design, and conducted primarily in India, China, Italy, Spain, and Nigeria. The total number of participants was 2852, with a median age of > 45 in all included studies. The stage of cancer varied among studies, with some studies including all stages (I-IV) while others included only early-stage or advanced-stage ovarian cancer. The included studies did not uniformly report details on lymph node and peritoneal involvement. Management included either surgery, chemotherapy, or both. The cut-off of SII ranged from 564.8 to 1000 among the studies. All studies mostly used the receiver operating characteristic (ROC) curve to determine the cut-off. Some studies used literature values or X-tile software to estimate the best cut-off. The median follow-up ranged from three to 126 months. Except for two studies, all studies reported multivariate adjusted outcomes. On assessment of study quality on the NOS scale, six studies received a score of eight, two received a score of seven, two received a score of six, and one received a score of nine (Supplementary File 3).

**Table 1:** Details of included studies

Study	Location	Sample size	Age	FIGO stage	Serous histology (%)	LN metastasis + ve (%)	Peritoneal involve-ment (%)	Treatment	Cut-off	Method for cut-off	Follow-up (months)	Out-comes	Adjusted outcomes	NOS score
Nie 2019 [18]	China	250	53	I-IV	71.6	50.8	38.4	Surgery	612	X-tile	46	OS, DFS	Yes	8
		283	54	I-IV	72.1	51.2	43.1	Surgery	612	X-tile	46	OS, DFS	Yes	8
Farolfi 2020 [29]	Italy	375	58	III-IV	42.1	NR	NR	Chemotherapy	730	Literature	NR	OS, DFS	Yes	9
Goenka 2022 [28]	India	49	NR	III-IV	NR	NR	NR	Chemotherapy	639	Median	3	OS, DFS	No	6
Liu 2020 [17]	China	108	57	II-IV	NR	NR	NR	Chemotherapy	701	ROC curve	NR	DFS	Yes	7
Ramon 2022 [27]	Spain	68	55.9	III-IV	82.3	NR	100	Surgery + chemotherapy	564.8	ROC curve	25	OS	Yes	8
Wang 2022 [26]	China	102	56	III	NR	NR	NR	Surgery + chemotherapy	872	ROC curve	36	OS, DFS	Yes	8
Bizzari 2023 [19]	Italy	359	54	I-III	39.8	18	NR	Surgery	1000	ROC curve	31	OS, DFS	Yes	7
Borella 2023 [25]	Italy	176	57	I	27	NR	NR	Surgery	730	Literature	126	DFS	Yes	8
Okunade 2023 [24]	Nigeria	91	47.6	I-IV	100	NR	39.6	Surgery + chemotherapy	610.2, 649	ROC curve	NR	OS, DFS	Yes	8
Song 2023 [20]	China	991	55	I-IV	83.6	55.3	NR	Surgery	727.9	ROC curve	NR	OS, DFS	No	6

*FIGO* International Federation of Gynecology and Obstetrics, *LN* Lymph node, *ROC* Receiver operating characteristic, *OS* Overall survival, *DFS* Disease free survival, *NOS* Newcastle Ottawa scale, *NR* Not reproted

### Meta-analysis

**Fig. 2:**
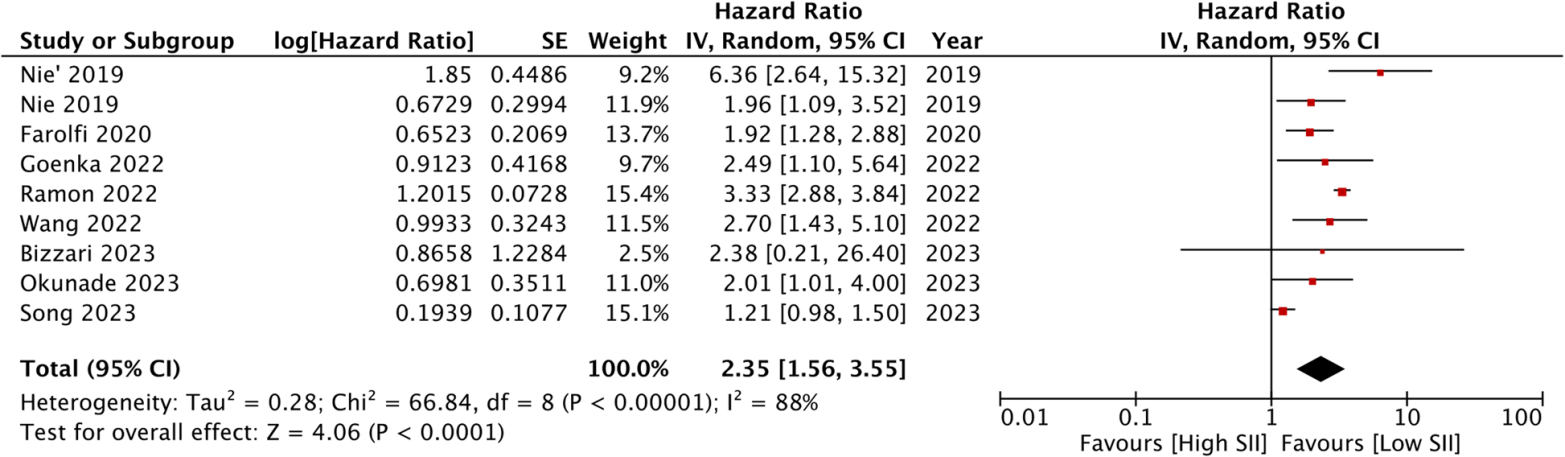
Meta-analysis of the association between high SII and poor OS in ovarian cancer. Red squares and horizontal lines in front of each study indicate the point estimates and 95% confidence intervals respectively. The star at the bottom of the figure denotes the pooled effect size

**Fig. 3:**
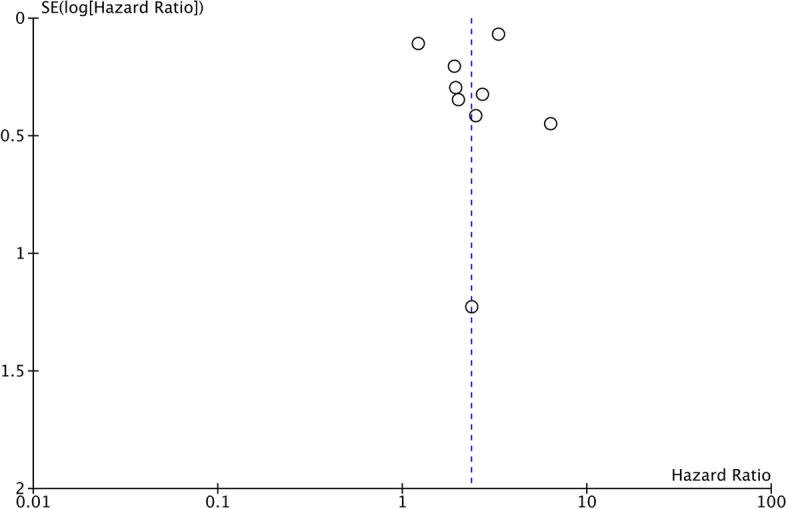
Funnel plot for the meta-analysis on OS. The position of the circles denote individual studies and their effect size in relation to the pooled effect size denoted by the dotted line

Nine cohorts from eight articles reported on the association between SII and OS in ovarian cancer. Pooled data showed that higher SII was a significant predictor of poor OS after ovarian cancer (HR: 2.35 95% CI: 1.56, 3.55 *I* ^*2*^ = 88%) (Fig. 2). The association remained significant after the sensitivity analysis. No asymmetry was detected on the funnel plot, indicating no evidence of publication bias (Fig. 3). Similarly, Egger’s test did not reveal publication bias (*p* = 0.82).

**Fig. 4:**
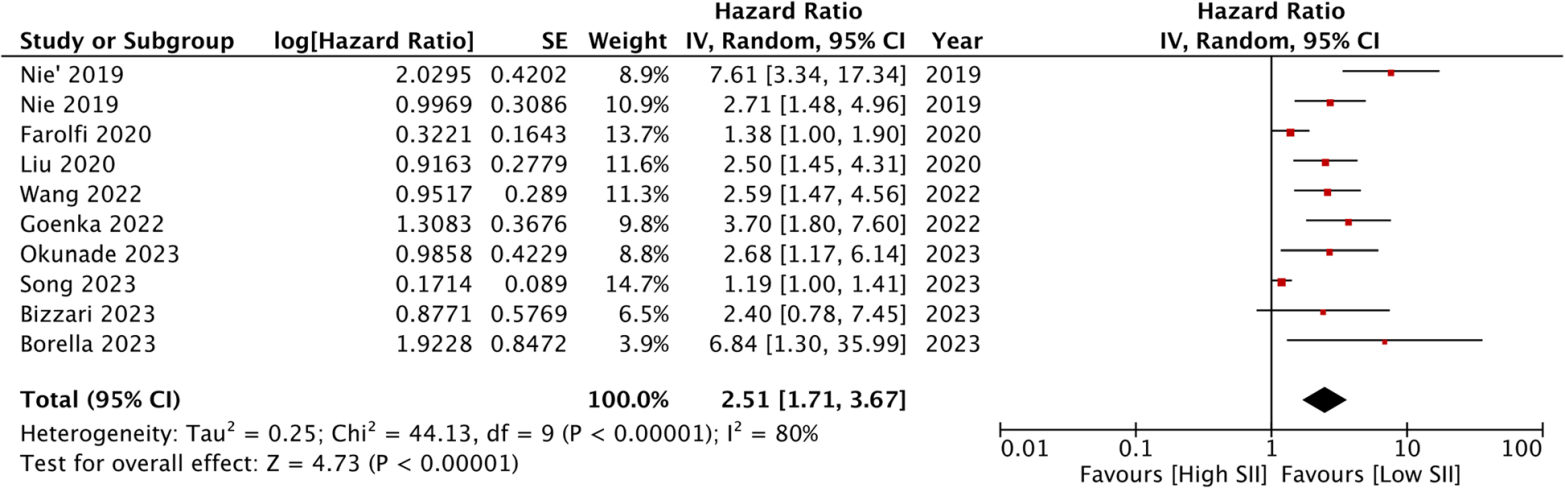
Meta-analysis of the association between high SII and poor DFS in ovarian cancer. Red squares and horizontal lines in front of each study indicate the point estimates and 95% confidence intervals respectively. The star at the bottom of the figure denotes the pooled effect size

**Fig. 5:**
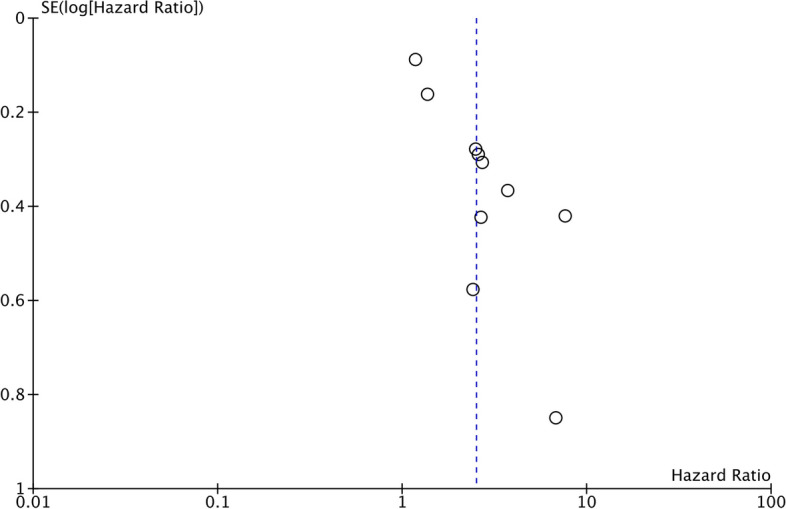
Funnel plot for the meta-analysis on DFS. The position of the circles denote individual studies and their effect size in relation to the pooled effect size denoted by the dotted line

Information on DFS was available from 11 cohorts. Meta-analysis showed that high SII was associated with worse DFS after ovarian cancer (HR: 2.51 95% CI: 1.71, 3.67 *I* ^*2*^ = 80%) (Fig. 4). The exclusion of any study during study during sensitivity analysis did not alter the significance of the results. There was no asymmetry on the funnel plot (Fig. 5), and Egger’s test did not reveal publication bias (*p* = 0.69).

Subgroup analysis was conducted for both OS and DFS based on study location (Asia, Europe, Africa), sample size (≥ 250, < 250 participants), FIGO stage (I-IV, III-IV), treatment (surgery, chemotherapy, surgery + chemotherapy), adjusted outcomes (yes, no), cut-off of SII (> 700, < 700), method of determining cut-off (ROC curve, X-tile, literature), and NOS score (8–9, 6–7). As shown in Table 2, the association between SII and OS/DFS remained statistically significant for most subgroup analyses. However, no significant association between high SII and OS was detected for studies reporting unadjusted data and with NOS scores of 6–7. Likewise, a non-significant association between SII and DFS was noted for studies from Europe, those reporting unadjusted data, and those using cut-off values from literature.

**Table 2:** Details of subgroup analysis

Variable	Groups	Cohorts	HR [95% CI]	I^2^
**OS**				
Region	Asia	5	2.33 [1.33, 4.07]	80
	Europe	3	2.62 [1.63, 4.22]	68
	Africa	1	2.01 [1.01, 4.00]	-
Sample size	≥ 250	5	2.05 [1.24, 3.39]	76
	< 250	4	3.21 [2.80, 3.67]	0
Stage	I-IV	4	2.14 [1.15, 3.96]	81
	III-IV	4	2.67 [1.94, 3.68]	56
Treatment	Surgery	4	2.24 [1.05, 4.76]	79
	Chemotherapy	2	2.02 [1.41, 2.91]	0
	Surgery + chemotherapy	3	3.12 [2.53, 3.84]	13
Adjusted data	Yes	7	2.66 [1.97, 3.57]	53
	No	2	1.55 [0.80, 3.03]	64
Cut-off	> 700	4	1.73 [1.13, 2.64]	64
	< 700	5	2.88 [2.07, 4.02]	46
Method of cut-off	ROC curve	5	2.16 [1.15, 4.08]	93
	X-tile	2	3.37 [1.07, 10.63]	79
	Literature	2	2.02 [1.41, 2.91]	0
NOS score	8–9	6	2.66 [1.95, 3.63]	61
	6–7	3	1.51 [0.90, 2.52]	34
**DFS**				
Region	Asia	6	2.73 [1.55, 4.82]	87
	Europe	3	2.10 [0.95, 4.62]	52
	Africa	1	2.68 [1.17, 6.14]	-
Sample size	≥ 250	5	2.13 [1.28, 3.55]	84
	< 250	5	2.85 [2.08, 3.89]	0
Stage	I-IV	4	2.69 [1.18, 6.15]	89
	III-IV	3	2.22 [1.21, 4.07]	75
Treatment	Surgery	5	2.98 [1.32, 6.76]	86
	Chemotherapy	3	2.19 [1.22, 3.95]	75
	Surgery + chemotherapy	2	2.62 [1.64, 4.18]	0
Adjusted data	Yes	8	2.71 [1.82, 4.05]	65
	No	2	1.98 [0.65, 6.01]	89
Cut-off	> 700	6	1.83 [1.27, 2.64]	70
	< 700	4	3.65 [2.33, 5.73]	34
Method of cut-off	ROC curve	5	2.04 [1.26, 3.31]	75
	X-tile	2	4.37 [1.59, 11.97]	75
	Literature	2	2.48 [0.55, 11.22]	71
NOS score	8–9	6	2.89 [1.70, 4.92]	74
	6–7	4	2.13 [1.14, 3.99]	81

*NOS *Newcastle Ottawa scale, *ROC *Receiver operating characteristic, *OS *Overall survival, *DFS *Disease free survival, *HR *Hazard ratio, *CI *Confidence intervals

## Discussion

Recently, the role of SII in predicting outcomes of oncological patients has become a focus of extensive research. [14–16] SII was identified as an easy and readily available marker that requires only platelet, neutrophil, and lymphocyte counts [13]. A review by Zhang et al. [30] demonstrated the ability of SII to predict both OS and DFS in patients with oral cancer, and showed that high SII was associated with 1.85- and 1.77-times higher risk of worse OS and DFS, respectively. A meta-analysis by Zeng et al. [31] found a similar predictive ability of SII for nasopharyngeal carcinoma. A study by Cheng et al. [32] collated data from 13 studies and showed that high SII is linked to a two-fold higher risk of worse OS and DFS in breast cancer patients. A study by Qiu et al. [33] that combined outcomes from eight cohorts noted that high SII was associated with worse OS in gastric cancer patients. Wang et al. [34] have found that urinary cancer patients with higher SII had poor OS, DFS, and cancer-specific survival. A review by Ji et al. [35] showed that SII can be a suitable marker for predicting OS and progression-free survival in patients with endometrial cancer. Individual cohort studies have also validated the prognostic ability of SII in cervical cancer [36, 37].

However, data on the prognostic value of SII in ovarian cancer has been scarce despite it being one of the most lethal malignancies in females. Previously, Mao et al. [21] analyzed data from six studies and showed that high SII was associated with poor OS (HR: 2.70, 95% CI: 1.98, 3.67) and poor PFS (HR: 2.71, 95% CI: 1.78, 4.12). However, in addition to the small number of the included studies, the quality of evidence was further impacted by including only partial data from the studies of Nie et al. [18] and Farolfi et al. [29]. A study by Nie et al. [18] reported separate outcomes for two cohorts (one training and one validation). Likewise, a study by Farolfi et al. [29] segregated the patient cohort into Platinum-resistant and Platinum sensitive groups and reported separate data for both groups. Only one group from each of these studies was included in the previous meta-analysis [21]. Further, in case of unavailable multivariate adjusted data [19], less preferable unadjusted univariate data were used. This study attempted to overcome these limitations and conducted an updated literature search to present the most reliable systematic review for the association between SII and survival after ovarian cancer.

In this study, a pooled analysis of 11 cohorts showed that patients with high SII had a 2.35 times worse OS and a 2.5 times increased risk of poor DFS. While the strength of the association varied between studies due to variations in sample size, cancer stage, and, most importantly, the SII cut-off, the pooled HR remained unchanged in statistical significance during sensitivity analysis, which demonstrates the robustness of the results. Publication bias was also assessed via visual inspection of funnel plots and Egger’s test. The lack of publication bias also adds to the credibility of available evidence. Nonetheless, this study detected high inter-study heterogeneity in the meta-analysis of both OS and DFS which is a significant limitation. We believe that such high inter-study heterogeneity could be explained by the differences in study locations, population characteristics, ethnicity, baseline comorbidities, cancer stage, treatment, and follow-up. Moreover, method of calculation of SII cut-off and the cut-off itself differed in the included studies. Therefore, a detailed subgroup analysis based on the maximum possible covariates was performed to overcome this potential limitation.

Numerous studies show that the survival of a patient with ovarian cancer is dependent on several factors like initial symptoms, age at diagnosis, cancer stage, level of cytoreduction, postoperative complications, pathological type, and adjuvant therapy [38, 39]. Therefore, assessing SII as a single factor in univariate analysis could lead to biased results. Multivariate adjustment of data potentially nullifies the effect of confounding factors and provides evidence on the ability of a factor to predict survival independently. [40]. No significant associations between high SII and OS/DFS were detected on subgroup analysis for studies reporting univariate data. In contrast, statistically significant associations were found in the subgroup of studies reporting adjusted data, cancer stage, and treatment. SII showed good predictive value for OS and DFS in studies reporting data of all cancer stages as well as those including only advanced ovarian cancer. Likewise, SII predicted outcomes in patients receiving surgery, chemotherapy, or both. While these results further confirm the value of SII in predicting outcomes in clinical practice irrespective of baseline characteristics, we were unable to determine the source of the high heterogeneity. The inter-study heterogeneity reduced to zero only in a few subgroup analyses with a small number of studies and remained high in others. This indicates that factors other than those included in the subgroups may be at play which need to be identified by further studies.

The SII cut-off to predict outcomes was another major source of interstudy variability. Cut-offs among studies varied widely between 564.8 to 1000. While subgroup analysis showed that cut-offs higher or lower than 700 were predictive of survival, the interstudy heterogeneity was not significantly reduced in either subgroups. One possible reason for this could be the significant variations in the SII cut-offs between studies as no three studies reported the same cut-off. We believe there is an urgent need to establish a specific universal cut-off for using SII in clinical practice [8, 9] [14–16]. Given the wide variability of cut-off values, further worldwide studies examining the predictive ability of different SII cut-offs are needed to identify the optimal cut-off.

SII is calculated using the neutrophil–lymphocyte and the platelet-lymphocyte ratio, which have both been shown to predict outcomes in ovarian cancer patients [41]. By combining the two markers, SII may exhibit improved prognostic ability compared to each variable alone. Neutrophils have been shown to facilitate tumor invasion, metastasis, and vascular proliferation using pro-inflammatory mediators like interleukins, vascular endothelial growth factor (VEGF), matrix metalloproteinases, elastases, and chemokines. Specifically, neutrophils cause the release of oncostatin M, which facilitates angiogenesis through the induction of VEGF [42]. Additionally, they release reactive oxygen species in the tumor microenvironment which promotes tumor growth by causing DNA and epithelial damage [43]. Transforming Growth Factor-Beta derived from the neutrophils has been shown to induce epithelial to mesenchymal transition, increasing cancer cells' invasiveness [44]. Additionally, neutrophils gather in premetastatic niches via CXCR2 or CXCR4-dependent mechanism and release oncostatin M, elastase, and S100A8/S100A9, which causes tumor cell proliferation [45]. They can inactivate T-cells, reduce cancer-related immunity by secreting substantial levels of reactive oxygen species, arginase, and nitric oxide, improve the penetrative function of tumor cells, and limit their immune surveillance by mobilization of H + -pump ATPase [45].

Similarly, platelet counts have been associated with cancer progression due to their ability to increase the epithelial-mesenchymal transition of tumor cells, aiding their motility, preventing apoptosis, and allowing tumor cell extravasation. [46, 47]. Cancer cells can promote tumor cell‐induced platelet aggregation by expressing several platelet receptors such as GPIb and GPIIbIIIa, which promote interactions with platelet bridging proteins von-Willebrand’s factor, fibronectin and fibrinogen. Such aggregation increases the metastatic potential of cancer cells by shielding cancer cells from physical stressors within the vasculature and by allowing evasion from the immune system’s effector cells [48, 49]. Moreover, activated platelets release platelet microparticles (PMPs) with membrane receptors and cytoplasmic constituents. Research demonstrated that proteins and chemokine receptors of PMPs can be transferred to malignant cells and enhance their invasiveness [50]. Platelets also enhance tumor angiogenesis by secretion of several proangiogenic factors like VEGF, platelet-derived growth factor, and epidermal growth factor [48].

Lymphocyte count is in the denominator in the calculation of SII, indicating an inverse relationship between lymphocytes and cancer progression. Indeed, lymphocytes are considered the cornerstone of defense against cancer cells. Cytotoxic T lymphocytes target cancer cells by identifying the mutated proteins and tumor antigens presented by the major histocompatibility complex on the tumor cell's surface. Helper T cells enhance the immune response triggered by cytotoxic T cells by secreting cytokines like interleukin-2, interferon gamma, and tumor necrosis factor-alpha. These cells also regulate the tumor microenvironment by counteracting the immunosuppressive mechanisms that cancer cells use by acting against regulatory T cells, myeloid-derived suppressor cells, and immune checkpoint proteins [51, 52]. B lymphocytes act via interferon-gamma and tumor necrosis factor-alpha to control cancer progression, while natural killer cells participate in tumor defense by attacking cancer cells without antigen activation [10].

This review has some limitations. All studies had a primarily retrospective design, which is prone to bias. Secondly, the number of included studies was limited. Information on important baseline variables, like lymph node metastasis, peritoneal involvement, etc., was not uniformly reported. This limited our ability to conduct a detailed subgroup analysis. Thirdly, most studies reported a mix of different cancer stages and treatments and did not segregate outcomes based on these variables. This precluded us from conducting separate analyses based on specific stages and treatment. Fourthly, we attempted to include adjusted data for most studies. However, some studies reported unadjusted data, which could have led to bias. Additionally, this review only analyzed OS and DFS. Other important oncological outcomes like disease control and objective response rates could not be assessed due to the lack of data from the included studies. Lastly, the included studies reported information from a limited number of countries. Further research is needed to improve the generalizability of the observations.

## Conclusions

SII can be a potential marker for predicting OS and DFS in ovarian cancer patients. High heterogeneity of the analysis is a significant limitation; hence, results should be interpreted with caution. Future, more extensive prospective studies from different regions of the world are needed to validate the results. Such studies should diligently report all baseline characteristics of included patients and segregate data on the prognostic ability of SII based on cancer stage and treatment protocols. Future studies should also take into account all known confounders influencing the survival rates of ovarian cancer patients. Lastly, research should also be directed towards identifying the most optimal cut-off of SII in predicting outcomes. Such data would aid in the routine clinical application of SII as a prognostic marker for ovarian cancer.

## Supplementary Information

Supplementary Material 1

Supplementary Material 2

Supplementary Material 3

## Data Availability

All data generated or analysed during this study are included in this published article and its supplementary information files.
