# Supplementary material for: Prognostic significance of systemic immune inflammation index for ovarian cancer: An updated systematic review and meta-analysis
Source: J Ovarian Res. 2025 Feb 27;18:41. doi: 10.1186/s13048-025-01626-1 (PMC11869409; doi:10.1186/s13048-025-01626-1)
Supplement: Supplementary file 1 — Supplementary Material 1 [file 13048_2025_1626_MOESM1_ESM.docx]

**Supplementary File 1: Search strategy**

**Embase**

1. ‘ovary'

2. ‘ovarian’

3. #1 OR #2

4. ‘cancer’

5. ‘malignancy’

6. 'carcinoma'

7. #4 OR #5 OR #6

8. ' systemic immune inflammation index’

9. ‘SII’

10. #8 OR #9

11. #3 AND #7 AND #10

**PubMed**

(((ovary) OR (ovarian)) AND (((cancer) OR (malignancy)) OR (carcinoma))) AND ((systemic immune inflammation index) OR (SII))

**Scopus**

( ovary OR ovarian ) AND ( TITLE-ABS-KEY-AUTH ( cancer OR malignancy OR carcinoma)) AND ( systemic immune inflammation index OR SII )

**Web of Science**

(((ovary) OR (ovarian)) AND (((cancer) OR (malignancy)) OR (carcinoma))) AND ((systemic immune inflammation index) OR (SII))

**CENTRAL**

[(ovary):ti,ab,kw OR (ovarian):ti,ab,kw] AND [(cancer):ti,ab,kw OR (carcinoma):ti,ab,kw OR (malignancy):ti,ab,kw] AND [(systemic immune inflammation index):ti,ab,kw OR (SII):ti,ab,kw]
