# Supplementary material for: Prognostic significance of systemic immune inflammation index for ovarian cancer: An updated systematic review and meta-analysis
Source: J Ovarian Res. 2025 Feb 27;18:41. doi: 10.1186/s13048-025-01626-1 (PMC11869409; doi:10.1186/s13048-025-01626-1)
Supplement: Supplementary file 2 — Supplementary Material 2 [file 13048_2025_1626_MOESM2_ESM.docx]

Supplementary File 2: List of excluded studies

| Excluded study | Reason |
| --- | --- |
| Feng 2023[1] | Not on SII |
| Huang 2021[2] | Not on SII |
| Espinos 2024[3] | Not on SII |
| Song 2023[4] | Not reporting survival data |
| Mleko 2023[5] | Not reporting survival data |
| Balescu 2024[6] | Not reporting effect size |
| Benvenuto 2020[7] | Not reporting effect size |

References

1. Feng J, Wang Q. Correlation of systemic immune-inflammatory response index with clinical data in patients with malignant ovarian tumor. Am J Transl Res. 2023;15:3309–17.

2. Huang H, Wu K, Chen L, Lin X. Study on the Application of Systemic Inflammation Response Index and Platelet-Lymphocyte Ratio in Ovarian Malignant Tumors. Int J Gen Med. 2021;14:10015–22. doi:10.2147/IJGM.S346610.

3. Espinós J, Aramendía JM, González-Martín A, Santisteban M, Sánchez L, Vizcay Á, et al. Prognostic value of systemic inflammation response indexes obtained from the complete blood count in patients treated for advanced ovarian carcinoma in front line. Clin Transl Oncol. 2024;26:3211–7. doi:10.1007/s12094-024-03523-3.

4. Song L, Qi J, Zhao J, Bai S, Wu Q, Xu R. Diagnostic value of CA125, HE4, and systemic immune-inflammation index in the preoperative investigation of ovarian masses. Medicine (Baltimore). 2023;102:e35240. doi:10.1097/MD.0000000000035240.

5. Mleko M, Pluta E, Pitynski K, Bodzek M, Kałamacki A, Kiprian D, et al. Trends in Systemic Inflammatory Reaction (SIR) during Paclitaxel and Carboplatin Chemotherapy in Women Suffering from Epithelial Ovarian Cancer. Cancers (Basel). 2023;15. doi:10.3390/cancers15143607.

6. Balescu I, Eftimie M, Petrea S, Diaconu C, Gaspar B, Pop L, et al. Prognostic Significance of Preoperative Inflammation Markers on the Long-Term Outcomes in Peritoneal Carcinomatosis from Ovarian Cancer. Cancers (Basel). 2024;16. doi:10.3390/cancers16020254.

7. Benvenuto G, Todeschini P, Paracchini L, Calura E, Fruscio R, Romani C, et al. Expression profiles of PRKG1, SDF2L1 and PPP1R12A are predictive and prognostic factors for therapy response and survival in high-grade serous ovarian cancer. Int J cancer. 2020;147:565–74. doi:10.1002/ijc.32935.
