# Supplementary material for: Prognostic significance of systemic immune inflammation index for ovarian cancer: An updated systematic review and meta-analysis
Source: J Ovarian Res. 2025 Feb 27;18:41. doi: 10.1186/s13048-025-01626-1 (PMC11869409; doi:10.1186/s13048-025-01626-1)
Supplement: Supplementary file 3 — Supplementary Material 3 [file 13048_2025_1626_MOESM3_ESM.docx]

Supplementary file 3: Quality assessment of included studies

| Study | Selection of groups | Comparability | Outcome assessment | NOS score |
| --- | --- | --- | --- | --- |
| Nie 2019[18] | 4 | 2 | 2 | 8 |
| Farolfi 2020[29] | 4 | 2 | 3 | 9 |
| Goenka 2022[28] | 4 | - | 3 | 7 |
| Liu 2020[17] | 4 | 2 | 1 | 7 |
| Bizzari 2023[19] | 4 | 2 | 1 | 7 |
| Borella 2023[25] | 4 | 2 | 2 | 8 |
| Ramon 2022[27] | 4 | 2 | 2 | 8 |
| Wang 2022[26] | 4 | 2 | 2 | 8 |
| Okunade 2023[24] | 4 | 2 | 2 | 8 |
| Song 2023[20] | 4 | - | 2 | 6 |

Numbers indicate the scores received by each study for each domain of the NOS score.

NOS, Newcastle Ottawa scale
